# Demand and supply factors of iron-folic acid supplementation and its association with anaemia in North Indian pregnant women

**DOI:** 10.1371/journal.pone.0210634

**Published:** 2019-01-30

**Authors:** Jithin Sam Varghese, Sumathi Swaminathan, Anura V. Kurpad, Tinku Thomas

**Affiliations:** 1 Division of Nutrition, St. John’s Research Institute, Bangalore, India; 2 Department of Physiology, St. John’s Medical College, Bangalore, India; 3 Department of Biostatistics, St. John’s Medical College, Bangalore, India; TNO, NETHERLANDS

## Abstract

Anaemia prevalence in pregnant women of India declined from 57.9% to 50.3% from National Family Health Survey (NFHS)-3 to NFHS-4. However, over the course of that decade, the uptake of iron and folic acid (IFA) supplementation for 100 days of pregnancy improved by only 15%. To understand demand side risk factors of anaemia specifically related to IFA intake, an in-depth survey was conducted on pregnant women (n = 436) in 50 villages and wards of Sirohi district of Rajasthan, India. At the demand side, consistent IFA consumption in the previous trimester was inversely and strongly associated with anaemia (OR: 0.26, 95% CI: 0.12, 0.55). Reasons for inconsistent consumption included not registering to antenatal clinic, not receiving IFA tablets from the health worker and perceived lack of need. At the supply side, an analysis of IFA stock data at various levels of the health care (n = 168) providers from primary to tertiary levels showed that 14 out of 52 villages surveyed did not have access to IFA tablets. The closest availability of an IFA tablet for 16 villages, was more than 5 km away. To improve the uptake of IFA supplementation and thereby reduce iron deficiency anaemia in pregnant women, a constant supply of IFA at the village or sub-centre level, where frontline workers can promote uptake, should be ensured.

## Introduction

Anaemia is characterised by low haemoglobin concentrations and could lead to adverse health outcomes such as maternal and peri-natal mortality and low birth weight [1,2]. In India, particularly among pregnant women, anaemia is a major public health problem, with the recently concluded nationally representative NFHS-4 [3] reporting a prevalence of 50.3%, with not much variation between rural (54.3%) and urban (50.9%) populations.

**Fig 1:**
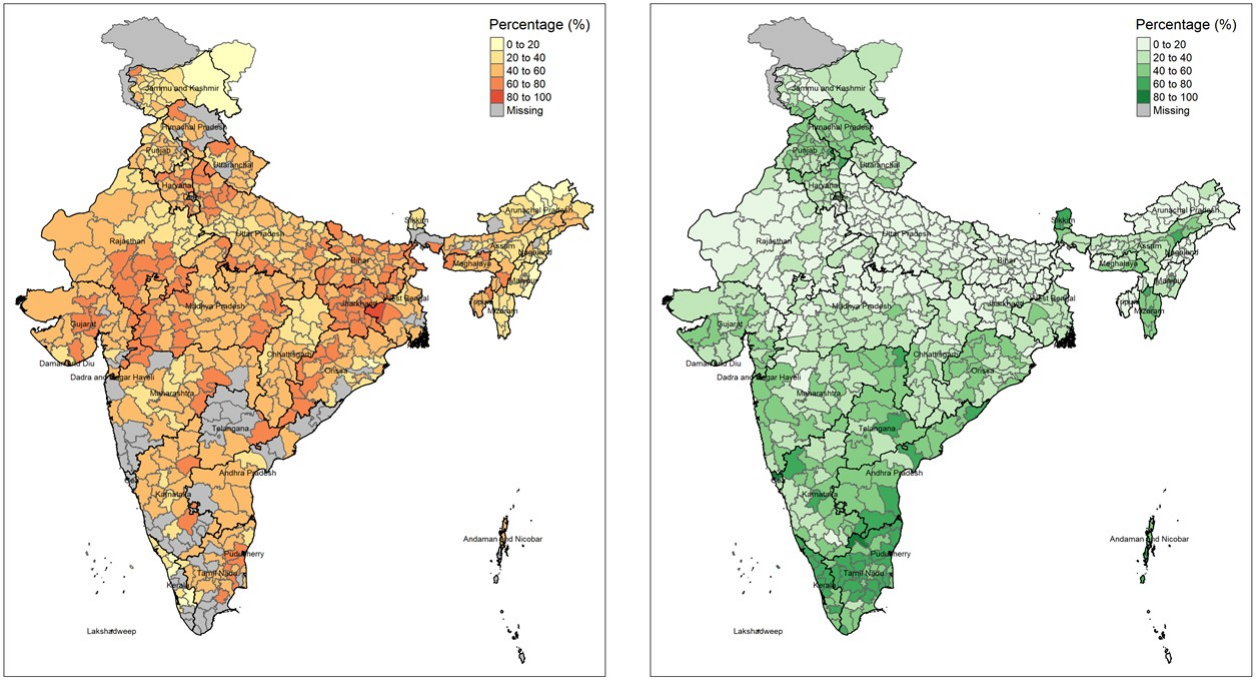
Findings from National Family Health Survey-4 related to anaemia in pregnant women. (A) Anaemia among Pregnant women and (B) percentage of women consuming 100 or more IFA tablets in India by district (NFHS-4). The maps were created from shapefiles available freely from datameet.org using tmap package (https://cran.r-project.org/web/packages/tmap/index.html) in R.

Iron deficiency is probably the most common cause of anaemia in India [4]. For this reason, under the National Iron Plus Initiative (NIPI, [5]), the government of India provides daily doses of IFA to pregnant women for a period of 100 days during their pregnancy. However, despite revisions to the NIPI programme with an increase in provision of elemental iron from 60 mg to 100 mg, the prevalence of anaemia in pregnant women has not come down significantly in the last 10 years between the nationally representative surveys, NFHS-3 conducted in 2005–2006 and NFHS-4 conducted between 2015–2016 among women aged 15–49 years. (Fig 1).

Plausible explanations for this continuing burden of anaemia, in spite of an apparently improved, but still not satisfactory compliance to the NIPI program as reported by NFHS surveys, are attributed to dietary and environmental reasons. The dietary reasons include an even lower than estimated iron density of diet, inhibition of iron supplement absorption due to diet components such as phytate and polyphenols [6,7] and a predominantly vegetarian diet leading to low intake compared to highly bioavailable heme iron [8–10]. The presence of the “leaky gut” syndrome [11,12] or environmental enteric dysfunction (EED) driven by water, sanitation and hygiene (WASH), hookworm infestation [13], and genetic causes [14,15] are other possible reasons.

An equally plausible set of reasons for the continuing prevalence of anaemia relates to the poor coverage of the IFA supplementation. This is expected to reach the pregnant women either through the antenatal services (ANC) or through frontline health workers. Although, the NFHS [3] surveys report an improvement in the uptake of IFA from 15.2% to 30.3% at a national level from round 3 to 4, it is far from being sufficient. There are both demand-side and supply-side problems contributing to the situation. There are important demand-side considerations, such as side effects, poor antenatal care utilization and forgetfulness, which are well known [16–18] and are held responsible for low compliance. However, there are little data on the supply-side deficiencies in an uncontrolled setting, such as relationship between antenatal care provision, health worker motivation [19] and availability of IFA stocks across different public health facilities [20,21], though periodical surveys such as the National Family Health Surveys tend to report some, but not all of these issues. An important logistic component relates to the distance to health facilities [22–24], that in turn determines health seeking behaviour. This has not been studied in any systematic way, to determine its impact. This paper studies the risk factors associated with anaemia and the coverage of the flagship IFA supplementation program (NIPI), among pregnant women in Sirohi district, Rajasthan, and maps the distance to the nearest iron supplements for women in the villages surveyed.

## Methods

An in-depth district level survey was conducted to collect data pertaining to both the demand-side from the perspective of the individual and the supply-side by examining facility level data. To study associated risk factors of anaemia at an individual level resulting from inadequate dietary intake and low compliance towards IFA tablets, data from survey, conducted in pregnant women in Sirohi District in Rajasthan state between March to June 2016, were used. This survey had collected detailed information on 5,324 households from 52 villages and wards, on maternal and child health in Sirohi, and aimed to identify the major risk factors of anaemia in children (0 to 59 months), adolescent girls (10 to 19 years), pregnant and lactating women, and stunting in children and adolescent girls. The survey also included a supply-side component which aimed to understand the functioning of the health system (from village to district level) in addressing malnutrition. All data other than the biomarkers were collected using questionnaires which were developed in consultation with experts and referring to literature on the current evidence available on nutrition specific and nutrition sensitive factors associated with malnutrition, anaemia and stunting, surveys conducted in India and elsewhere, and health system guidelines. The questionnaires were pre-tested and translated to local language before being used in the field.

### Ethical approval

Ethical approval was obtained from the institutional ethics committee of the St John’s Medical College and informed written consent was obtained from each of the survey respondents both from the demand and the supply side. Permission from the state health department and the office of the district collector were procured prior to commencement of the survey.

### Survey design

A multi-stage cluster sampling of Primary Sampling Units (PSU) which were villages (for rural areas) and wards (for urban areas) as per the Census of India [25] was employed. The sampling was stratified by block, rural-urban status and the PSUs were selected by probability proportional to size sampling. The definition of a village in rural areas corresponded to geographically defined villages, that relate to meaningful social and administrative divisions; in urban areas, communities are based on well-defined wards and are considered to be demographically homogeneous [26]. The eligible households were randomly sampled in the next stage of sampling for the demand survey. For the supply side component of the survey, the facilities catering to the sampled villages were surveyed. Geographical Information System (GIS) mapping of each village/ward was done. A village representative provided information on distance of village from different health facilities such as Sub Centre, Primary Health Centre (PHC), Community Health Centre (CHC), Sub Divisional Hospital, District Hospital and Private Clinics, and health workers such as Accredited Social Health Activists (ASHA) and Anganwadi Workers (AWW). ASHA workers report to an Auxiliary Nurse Midwife (AN) located at the Sub Centre. In Sirohi, six Sub Centres report administratively to either a PHC or a CHC, which then report to the District Hospital. Village level information on items such as local agricultural production, waste disposal, industrial emissions etc. were also collected from the Panchayat head or any senior literate and knowledgeable member of a village or ward.

### Household information (Demand)

Household information on possessions, food security and details on education, age, occupation and marital status of members were collected from the head or a responsible adult member in the household. Data pertaining to medical information and diet of pregnant women collected in the survey was considered for this analysis. Trained field staff conducted interviews and anthropometric measurements of height and weight in all eligible individuals. Haemoglobin (Hb) was measured from capillary blood samples using Hemocue 301 analyzer in all sampled pregnant women. Pregnant women with Hb levels below 110g/l [27] were classified as anaemic for this analysis. The survey covered 454 pregnant women in Sirohi district, Rajasthan. Out of these, data on 436 subjects who had Hb measurements were considered for this analysis. There was no altitude adjustment of Hb required. Reported symptoms related to anaemia such as fatigue, were collected. IFA demand was measured in terms of receipt and consumption of IFA tablets for each trimester up to the trimester when a pregnant woman was recruited. Diet diversity was measured in terms of the food group consumed and had 11 categories of food groups. Additionally, frequency of consumption of vitamin A and vitamin C rich foods on a monthly basis was also collected. GIS data (Latitude, Longitude and Altitude) for households was measured using Samsung Tab 4—T231 (Samsung Electronics), an Android device commonly used in surveys.

### Facility information (Supply)

To get the perspective from the supply side, health care personnel from 168 facilities (70 Accredited Social Health Activists or ASHAs, 52 Anganwadi workers, 30 Sub Centres, 20 Primary Health Centres, 6 Community Health Centres) in Sirohi catering to the villages/wards that were sampled were interviewed. In addition, specific questions on IFA supply was recorded at these centres, both by recording responses from health care providers as well as through observations by field workers of drug kits provided for ASHAs and AWW, and through stock checks in the pharmacy for Sub Centres and Primary Health Centres. GIS data for frontline workers (FLWs) in a village was assumed to be centroid of surveyed households in the village. GIS data for facilities was assumed to be location of village/town where the facility was housed, as this information for facilities was not collected. The distance to the nearest IFA tablet was the distance to the closest health facility which had IFA stocks had the sampled village in its catchment area.

### Statistical analysis

Descriptive statistics were reported as numbers and percentage for categorical data and mean ± standard deviation for continuous normally distributed data. In the individual dataset, the factors associated with anaemia were analysed using chi square test, independent sample t test and Mann-Whitney U Test as appropriate. The significant factors from these analyses were used in bivariate and multiple variable logistic regression of anaemia. The factors considered for analysis in individual data sets were dietary diversity, health service utilization, morbidity, usage of intoxicant substances like paan (betel leaf, arecanut) and tobacco, water, sanitation and hygiene practices. Mobile usage was considered as a proxy for woman’s economic status. The factors were considered in the multiple variable analysis with a cut off value of p < 0.2 in bivariate logistic regression. The final model in the multiple variable analysis was identified using a hierarchical iterative process. Confounding and interaction effects were also explored. All analyses were performed using R software (https://cran.r-project.org/bin/windows/base/old/3.3.3/) and survey weighted logistic regression was carried with R survey package [28].

## Results and discussion

### Results

The survey covered a sample of 454 pregnant women of which haemoglobin was measured for 436 (4% non-respondents). There were 110, 187 and 139 pregnant women in their first, second and third trimesters respectively out of the 436, at the time of survey. A detailed description of the study population is provided in Table 1.

**Table 1:** Socio-demographic and economic characteristics of pregnant women in Sirohi district, India.

	N (%)
**Number of Pregnant women interviewed**	454
Rural	346 (76)
Urban	108 (24)
**Number of Pregnant women for whom Hb was measured**	436 (96)
Rural	334 (97)
Urban	102 (94)
**Trimester**	
First	110 (25)
Second	187 (43)
Third	139 (32)
**Age of household head**^#^	39.6 ± 13.9
**Educational level of household head (%)**	
Illiterate	47.9
Primary (1st to 5th grade)	16.5
**Type of family**	
Nuclear	258 (59)
Extended	47 (11)
Joint	131 (30)
**Main source of income**	
Agriculture	55 (13)
Non-agriculture	265 (61)
Both	116 (27)
Household size^#^	5.6 ± 2.2
**Socio-demographic characteristics of pregnant women**	
Age (yrs.) ^#^	24.9 ± 4.6
Literate	169 (39)
Work for income	111 (25)
Read state language	140 (32)
Write state language	133 (31)
Currently married	429 (98)
Age at marriage > = 18 years	274 (63)
Multiparous	203 (47)
Use mosquito net	228 (52)
Suffer from malaria in last three months	16 (4)
Use mobile phone	266 (61)

^#^- Mean ± SD

While 66% of the women were anaemic, 35% were moderately or severely anaemic. General fatigue was the most reported anaemia related symptom among the anaemic women (59%) followed by giddiness, dizziness or headache. Among 299 anaemic women, 37 (12%) did not report any symptom and out of these 17 (6%) were mildly anaemic. Among 436 pregnant women, 303 (69%) reported registering for antenatal care, although the ANC card was available with only 209 (48%) of the respondents. Of the 227 with whom the card was not available, 66 (29%) were in their third trimester and 88 (39%) in the second trimester. About 59% of pregnant women chose to register at a government facility. Those who registered elsewhere (10%) cited quality of care, higher trust of non-government facilities and distance to government health centre as major reasons for not registering at a government centre. More information on utilization is available in Table 2.

**Table 2:** Antenatal care and morbidity of pregnant women in Sirohi district, India (N = 436).

Antenatal services received by pregnant women	N (%)
Received antenatal check-up	208 (48)
Physical Examination	65 (15)
Measurement of weight	62 (14)
BP measured	59 (13)
Received TT vaccination	48 (11)
Consumed IFA in previous month’s trimester^	155 (36)
Folic Acid received in 1st trimester as per ANC card^#^	4 (4)
Folic Acid consumed in 1st trimester (previous month's trimester = 1)^^#^	30 (20)
IFA received in 2nd or 3rd trimester* as per ANC card	47 (14)
IFA consumed in 2nd/3rd trimester* (previous month's trimester = 2 or 3; N = 270)	125 (46)
Ever Received deworming tablet as per ANC card during pregnancy	11 (2)
Received professional breastfeeding counselling as per ANC card	14 (4)
Hb examined at health centre as per ANC card	71 (29)
**Prevalence of Anaemia**	299 (69)
Mild anaemia	146 (34)
Moderate anaemia	140 (32)
Severe anaemia	13 (3)
**Morbidity (last one month)**	
Diarrhoea	64 (15)
Vomiting	204 (47)
Fever	117 (27)
**Symptoms related to anaemia**	
General fatigue	259 (59)
Breathlessness on routine and somewhat strenuous work	156 (36)
Giddiness, dizziness, headache	197 (45)
Appetite loss	125 (29)
Weight loss	57 (13)
Blurring of vision	149 (34)
Sudden swelling of feet	68 (16)

^—calculation does not include those women (N = 13) in their first month of pregnancy;

^#^—Folic Acid is provided in Trimester 1;

*—IFA is provided in Trimester 2 and 3

### Factors associated with anaemia in pregnant women

The factors associated with anaemia in pregnancy in the bivariate and multiple variable logistic regression analyses are presented in Table 3. The final logistic regression model showed IFA supplementation to be negatively associated with anaemia such that there was 74% lower odds of anaemia with consistent consumption of IFA or folic acid supplement in previous trimester (OR: 0.26, 95% CI: 0.12 to 0.55, p = 0.001). In addition, month of pregnancy (OR: 1.21, p = 0.012), mobile usage (OR: 0.41, p = 0.010), usage of intoxicant substances (OR: 2.27, p = 0.075), number of women in household (OR: 0.72, p = 0.079) were also significantly associated with anaemia (Table 3).

**Table 3:** Individual and household characteristics associated with anaemia outcome in pregnant women of Sirohi district, India.

	*Unadjusted*		*Adjusted*	
Individual characteristics	Odds Ratio	P-value	Odds Ratio	P-value
Month of Pregnancy	1.13	0.07	1.211	0.012
Previous month's trimester	1.58	0.05		
Writing state language	0.49	0.09		
Mobile usage	0.29	<0.001	0.409	0.010
Low BMI	2.04	0.04		
Usage of tobacco	3.20	0.03	2.268	0.075
Past history of seizures	2.45	0.06		
Awareness of health and nutrition programs	0.95	0.08		
Receipt of blood transfusion in last 1 year	9.74	0.03		
**Dietary consumption**				
vegetables and pulses	0.62	0.02		
Guava	1.33	0.09		
Jackfruit	0.63	0.02		
Papaya	1.69	0.05		
Pumpkin	0.02	0.04		
Avoid meat	9.10	0.03		
Avoid poultry	23.31	0.01		
Avoid fish	4.55	0.09		
Avoid eggs	4.61	0.10		
**ICDS**^a^				
Consuming ICDS Supplementary Nutrition alone	2.82	0.05		
Consuming ICDS Supplementary Nutrition completely	2.49	0.10		
**Antenatal Care**				
Consistent IFA/FA consumed (every day in last month's trimester)	0.29	<0.001	0.258	0.001
Never Received IFA tablets	1.64	0.06		
ANC Check-up in 3rd Trimester	0.89	0.04		
Mobile Registration for ANC- Registered vs Not registered	4.17	0.01		
**WASH**				
General use of soap	0.19	0.05		
Brushing teeth at least once a day	0.35	0.06		
Health seeking behaviour index	0.86	0.07		
**Household Characteristics**				
Altitude	0.99	0.07		
Total Household Members	0.91	0.05		
Number of women in household	0.70	0.04	0.724	0.079
Relationship to head of household	6.01	0.06		
Non-Hindu religion	6.13	0.05		
Wealth Index	0.88	0.02		
Wealth Quintile	0.83	<0.001		
NREGA beneficiary	2.42	0.05		
Expected number of pregnant women in village			0.994	0.575

^a^ICDS- Integrated Child Development Services scheme. Odds ratio from survey weighted logistic regression analysis. Adjusted odds ratio after including all variables listed in the table.

### Factors associated with IFA consumption in pregnant women

**Fig 2:**
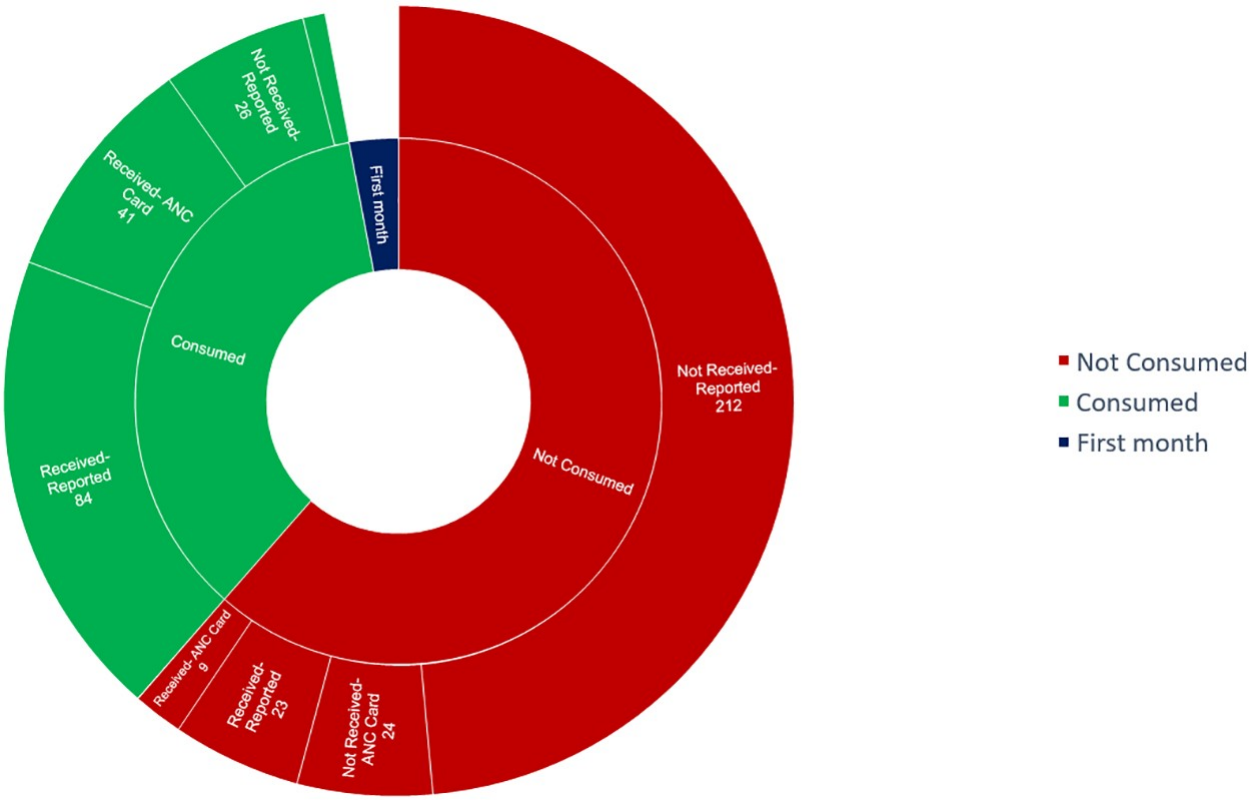
Reporting of consumption and receipt of iron and folic acid supplements. Values are number of subjects.

Since consistent consumption of iron and folic acid or folic acid tablets is associated with anaemia, the timely receipt of IFA supplements by pregnant women in Sirohi is critical and is examined in Fig 2. Among those who did not receive IFA, ‘no provision’ from the health worker was the most common reason. Among those who received supplements and did not consume them, low palatability, unrealized need, constipation and forgetfulness were the major reasons.

ANC registration was significantly associated with consumption of IFA or folic acid supplements by pregnant women in the previous month’s trimester (χ^2^ = 69.7, p<0.01, N = 423). Majority of those who did not consume did not receive IFA (88%). Table 4 provides a summary of ANC registration, IFA supplementation status and health worker visit. There was no association between health worker visit and receiving IFA (χ ^2^ = 2.8, p>0.05, N = 326) in Trimester 2 and 3. A health worker was more likely to visit those pregnant women who were registered under ANC (χ ^2^ = 29.7, p<0.01, N = 436).

**Table 4:** Antenatal care registration and health worker visits in surveyed population (N = 436).

	Health worker visits regularly	Health worker does not visit
**ANC Registered**	**135**	**168**
Did not receive IFA (Trimester 1*)	19 (14%)	20 (12%)
Received IFA (Trimester 2 and 3)	24 (18%)	23 (14%)
Did not receive IFA (Trimester 2 and 3)	92 (68%)	125 (74%)
**ANC Not Registered**	**23**	**110**
Did not receive IFA (Trimester 1*)	12 (52%)	59 (54%)
Received IFA (Trimester 2 and 3)	0 (0%)	0 (0%)
Did not receive IFA (Trimester 2 and 3)	11 (48%)	51 (46%)

*Eligible for folic acid; IFA- Iron and Folic Acid

### Availability of IFA to pregnant women

Fourteen of 52 surveyed villages in Sirohi did not have access to an IFA tablet except at the CHC or District Hospital level. Nineteen villages had access at the ASHA or Anganwadi level, while 9 and 10 villages had access at the sub-centre and PHC level respectively. Distance between two geographic locations, also known as the geodesic, was calculated as the shortest distance between two points on an ellipsoid, namely WGS84 [29]. Reported distance to each type of facility, per information from the village representative, was also recorded.

**Fig 3:**
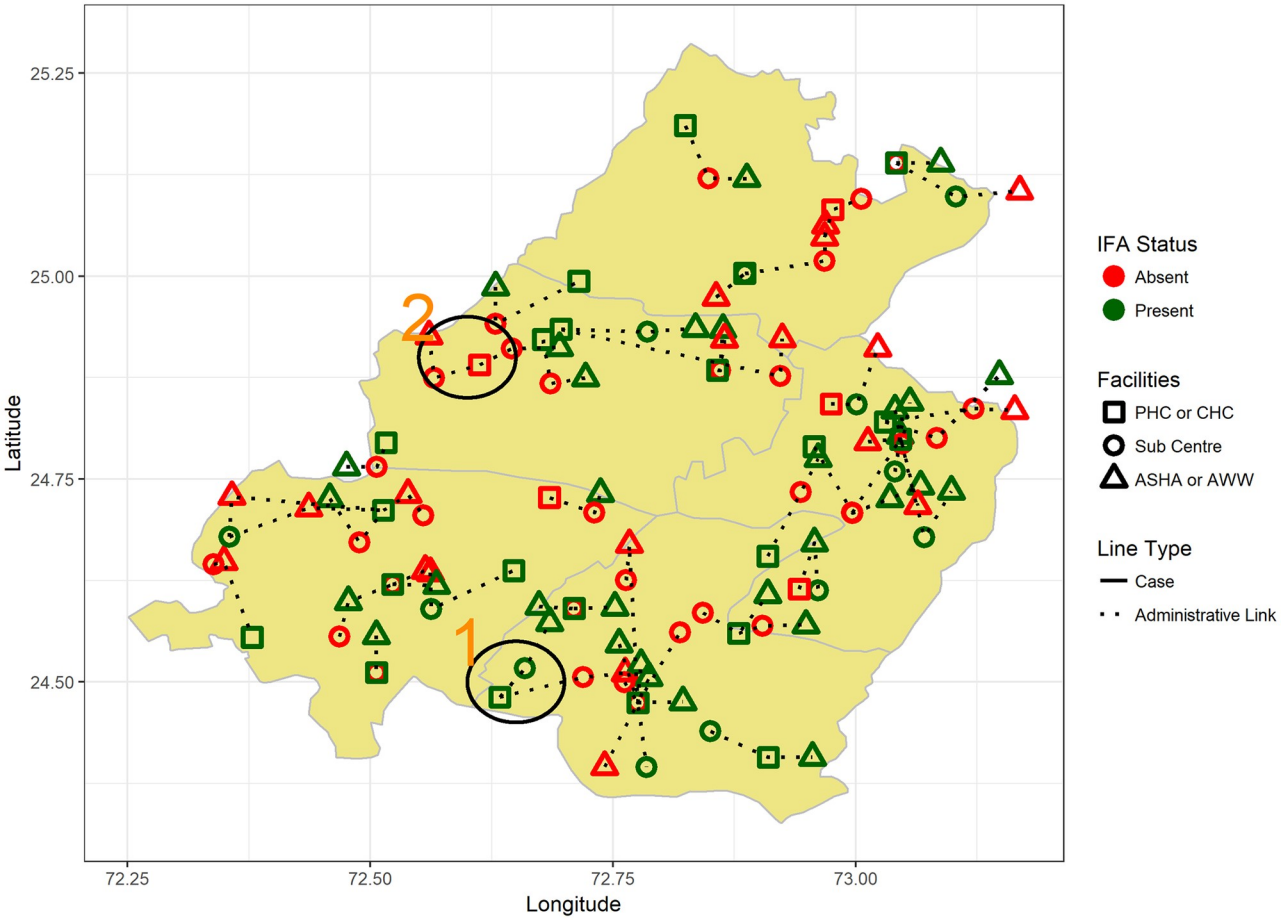
Geographical distribution of health facilities, inter-facility relationships and availability of IFA supplementation. The polygon was filtered from the shape file and modified using ggplot2 package (https://cran.r-project.org/web/packages/ggplot2/index.html) in R.

There are 4 villages which had IFA availability only at the District Hospital level (as per referral structure) which was 31.5 km (median reported distance) from the villages. On the other end, among the remaining villages 17 had IFA tablet available with either the ASHA or Anganwadi worker within the village at geodesic distance 0.6 km [IQR: 0,5.5], while the reported median distance was 0 km [IQR: 0, 7.2]. Excluding the farthest and nearest availability of IFA, for the remaining villages the nearest tablet was at geodesic distance of 3.6 km [IQR: 1.2, 8.5] while the reported distance was 5 km [IQR: 0.5, 10]. For 15 villages, the nearest IFA tablets were more than 5 km away (geodesic) from the source. Fig 3 represents the location of different facilities, administrative relationships between the facilities and the status of their IFA stock spatially. Table 5 contains information on health facilities with IFA supplements and their proximity to villages.

**Table 5:** Distance from different levels of public health facilities where IFA is available to surveyed villages.

IFA Available with	N	Geodesic distance (km)	Reported distance (km)
**Asha**	10	0 (Within village)	0 (Within village)
**Anganwadi**	7	0 (Within village)	0 (Within village)
**Sub Centre**	8	1.06 (IQR: 0.5 to 2.5)	0.5 (IQR: 0 to 3.5)
**PHC**	10	7.9 (IQR: 4.4 to 8.9)	10 (IQR: 7.3 to 11.8)
**CHC**	9	2.2 (IQR: 1.2 to 8.7)	2.5 (IQR: 0 to 10)
**District Hospital**	4	22.6 (IQR: 16.2 to 24.3)	31.5 (IQR: 22.25 to 38.75)

IQR: Inter Quartile Range

Fig 3 provides administrative linkages between different health facilities and the status of their IFA stocks. We randomly shifted the location of the village to North, South, East or West by 5km for confidentiality purposes. “1” refers to an ideal case where all facilities from village level onwards have IFA stocks. “2” refers to a non-ideal case where none of the facilities, except at the district level have IFA stocks. Approximately 50% of villages do not have IFA stocks below or at Sub-Centre level.

Both geodesic (r = -0.21, p = 0.15) and reported distance (r = -0.18, p = 0.23) to the health facility with IFA supplements were negatively (but not significantly) correlated with the proportion of women receiving IFA supplementation in the village.

### Discussion

The burden of anaemia was high at 66% in pregnant women of Sirohi district and consistent consumption of IFA/folic acid supplements during the previous trimester of pregnancy was the single most strongly inversely associated factor for anaemia. In addition low mobile usage, low number of women in household, use of tobacco and paan, late month of pregnancy increased the odds of anaemia. Of these, mobile usage (possibly a proxy for latent unmeasured variables such as woman’s empowerment and access to information in addition to woman’s economic status), substance use and IFA/folic acid intake are factors amenable to intervention.

Poor consumption of IFA supplements is a key risk factor contributing to anaemia among pregnant women in Sirohi. Though anaemia is a complex and multi-faceted problem which involves interaction of various nutrients, iron supplementation could alleviate a considerable proportion [30] of anaemia in India. As observed in Sirohi, a low intake of IFA supplements resulting from a combined effect of low stocks and poor demand drives anaemia among pregnant women. There was no significant difference of IFA receipt between women those who were visited by a health worker and those who were not. A possible explanation for this could be low stocks of IFA with frontline workers and IFA supply only from centralized locations such as Sub-centres or Anganwadi centres and not at homes. It could also mean that the health worker responsible for distributing IFA tablets was not the one who visited pregnant women at their homes. However, other studies have shown that antenatal care utilization can be improved from health worker visits [31]. The present study also shows that health worker visits are more likely for those women who are registered for antenatal care.

It is important to note that more than one-fourth of the villages surveyed did not have access to IFA supplements within a 5 km radius. As per Fig 2, most women did not consume IFA supplements not because of perceived side-effects but because they were not provided the same. NFHS-4 reports only 25.9% of pregnant women in rural areas consume 100 IFA tablets during pregnancy. Other studies in South Asia have reported that geographic proximity to health service is important in its utilization, especially for antenatal care, chronic diseases and infectious diseases [22,23]. To ensure stocks are in place in all health facilities, the local government could re-evaluate the supply chain management information system in place as well as provide for other measures such as buffer stocks and pharmacy budgets for health facilities. If so, health workers could plan the effective generation of demand in the community. For this, at least two actions are required; that the supply of IFA should be proactive and constant at the village level or the sub-centre, and that the distance to the nearest tablet should be at least at the Sub Centre level if not at the village level ensuring that visiting health workers (ANMs, ASHAs) have stock and would be able to supply pregnant women with IFA tablets.

Anaemia as a result of use of substances such as paan (betel leaf and/or betel nut) or tobacco is well-known [32,33]. Almost 40 (9%) pregnant women consumed paan masala or chewable tobacco on a routine basis. Smokeless tobacco [32] during pregnancy is observed to decrease haemoglobin levels, similar to cigarette smoking. In addition to reducing maternal red blood cell count, the habit can also lead to decreased serum Vitamin B_12_ and maternal red blood cell folate. The increasing odds of anaemia with month of pregnancy is a well-known phenomenon, often referred to as the physiological anaemia of pregnancy [34].

This analysis is not without limitations. Firstly, we surveyed only 52 PSUs (villages and wards) of which, reported distance to health facilities were available only for 48. However, the PSUs were sampled based on a probability proportional to size (PPS) design and ought to be representative of the district. Secondly, access to different health facilities were not considered when estimating the distance to the nearest IFA supplement. It is possible [35,36] that a health facility is close to a village, but not accessible due to socio-cultural reasons. Thirdly, distance and receipt of IFA supplementation were not significantly associated at a village level. As a result, the optimal location of the health facility for optimal provision of IFA cannot be defined. Fourth, since the road networks in the rural part of the district is poor, geodesic might not convey the true picture of distance. We tried to overcome this limitation by using the reported distance to the health facility as reported by the village representative along with the geodesic. Finally, it is possible that the stock-out of IFA which our team observed (we did not assess folic acid stocks) prevailed only for the duration of the survey period. This limitation is common to all cross-sectional studies.

## Conclusion

The prevalence of anaemia among pregnant women in Sirohi was higher than the national prevalence. Anaemia in pregnant women was associated with inconsistent consumption of IFA/FA. IFA not supplied by health worker was identified as demand side factor associated with inconsistent IFA consumption. With more than half the sample of pregnant women not being visited by health worker during pregnancy, it is critical that IFA is available at the nearest health facility. However, it was observed that about 50% of the villages did not have IFA available at the nearest health facility. While anaemia among pregnant Indian women continues to be a priority issue for the government, focussed interventions such as regular haemoglobin testing and ensuring successful implementation of the National Iron Plus Initiative nuanced for the local context and access to IFA can go a long way in meeting anaemia reduction targets in India.
